# Venous Thromboembolism Prophylaxis in Patients Treated for Acute Lymphoblastic Leukemia: A Comprehensive Systematic Review and Meta-Analysis

**DOI:** 10.7759/cureus.70078

**Published:** 2024-09-24

**Authors:** Bareq S Al Lami, Shad B Aziz, Yousif N Al-Tawil, Rawen Aras, Blnd D Dlshad, Rose Wilya, Hanan Slevanay, Zahraa Sarkawt, Taha Fadhel, Avin Salahaddin, Lazha Abdulla, Gunai Hussein, Vena Abdulwahhab, Hivi Albarznji

**Affiliations:** 1 General Medicine, Hawler Medical University, Erbil, IRQ; 2 College of Medicine, Hawler Medical University, Erbil, IRQ

**Keywords:** all, antiocoagulation, l-asparaginase, malignancy, thrombophylaxis, vte

## Abstract

Acute lymphoblastic leukemia (ALL) is a common malignancy in children, often treated with intensive chemotherapy regimens. Venous thromboembolism (VTE) poses a significant risk during ALL treatment, leading to suboptimal outcomes. Thromboprophylaxis is crucial in mitigating this risk, but its efficacy and safety remain uncertain. This systematic review and meta-analysis aimed to evaluate the effectiveness of thromboprophylaxis in reducing VTE incidence during ALL treatment, focusing on antithrombin, apixaban, and enoxaparin. A systematic literature search adhering to the Preferred Reporting Items for Systematic Reviews and Meta-Analyses (PRISMA) guidelines was performed. Randomized controlled trials (RCTs) investigating thromboprophylaxis in ALL were included. Data extraction and quality appraisal were performed independently by three authors. Meta-analysis was conducted using Review Manager software. Three RCTs met the inclusion criteria. Apixaban, enoxaparin, and antithrombin were assessed in these trials. Meta-analysis revealed significantly reduced odds of VTE with thromboprophylaxis compared to standard care (odds ratio (OR): 0.47, 95% confidence interval (CI) 0.29-0.75; relative risk (RR): 0.52, 95% CI 0.33-0.83). However, no significant difference in bleeding risk was observed (OR: 1.33, 95% CI 0.42-4.21; RR: 1.32, 95% CI 0.43-4.07). Heterogeneity among studies was moderate. This study showed that thromboprophylaxis with apixaban, enoxaparin, or antithrombin significantly reduces VTE incidence during ALL treatment. Despite some limitations, including heterogeneity and potential biases, these findings support the adoption of tailored thromboprophylaxis strategies to improve outcomes in ALL patients. Further research is warranted to optimize these approaches and address remaining uncertainties.

## Introduction and background

Acute lymphoblastic leukemia (ALL) is a malignancy of the lymphoid cell lineage characterized by the uncontrolled and rapid proliferation of lymphoid progenitor cells known as lymphoblasts, which are present in the bone marrow and peripheral blood. Although ALL can occur in adults and has a small peak occurring at the age of 50, it is predominantly a cancer of childhood accounting for 23% of all childhood malignancies under 15 years of age, making it the most common malignancy in children. Nearly 60% of ALL cases are diagnosed before the age of 20 years with a peak incidence between the ages of one and four years [1,2].

One of the major adverse events that can take place when treating a patient with ALL is venous thromboembolism (VTE), and the associated complications lead to suboptimal ALL treatment due to premature therapy discontinuation, therapy interruptions or loss of central venous catheters, and hence decreased treatment outcome, reflected in inferior complete remission or overall survival rates. Although direct mortality is infrequent [3], patients with VTE frequently endure long‐term morbidity.

There’s a high incidence of VTE in ALL patients (compared to other types of malignancies) who are undergoing chemotherapy, mostly due to the use of L-asparaginase and steroids [4]. L-asparaginase successfully eliminates human lymphocytes by reducing the level of circulating asparagine; however, it also disrupts the production and accelerates the elimination of anti-clotting proteins, namely, antithrombin III, thus causing disruptions in blood coagulation, which in turn plays a role in the development of VTEs. In addition, cancer cells can produce procoagulant, fibrinolytic, and inflammatory molecules, thereby inducing a hypercoagulable state. Moreover, cancerous cells can play a role in activating platelets and endothelial lining cells, which in turn can result in the down‐regulation process of anticoagulants and upregulation of procoagulants [5]. Despite ALL being a malignancy that is more commonly seen in children, VTE occurrence has a higher incidence in adult patients with ALL [6].

This systematic review and meta-analysis aim to address the current gap in the studies with regard to the prevention of VTE in patients being treated with ALL, with our primary focus being the prophylactic intervention, diagnosis, and supervision of patients with ALL and the mitigation of risk to all those with the likelihood of developing the damaging and potentially fatal side effects of the chemotherapeutic treatment. The primary objectives of this study include the following: First, we aim to investigate whether thromboprophylaxis is effective in reducing the incidence of VTE during therapy in ALL treatment for pediatric and adult patients. In addition, we aim to investigate the adverse effects of thromboprophylaxis measures during therapy in ALL treatment and to see which thromboprophylaxis regimen has the best efficacy in reducing the VTE incidence in ALL treatment.

## Review

Methods

Search Strategy and Data Sources

We conducted a systematic review according to the Preferred Reporting Items for Systematic Reviews and Meta-Analyses (PRISMA) guidelines [7]. Electronic databases, including Clinicaltrials.gov, Google Scholar, MEDLINE (PubMed), and the Cochrane Library, served as sources for our comprehensive literature search. A combination of Medical Subject Headings (MeSH) and Boolean operators were used to maximize the inclusivity of the search terms. Synonymous and variant expressions related to ALL, VTE, and prophylactic anticoagulation strategies were included. All possible articles found by using these electronic database searches were screened. Manual searches of reference lists complemented electronic database searches. Three authors independently reviewed and assessed the text of the articles that were deemed potentially relevant to the main topic. After that, full papers were identified and were further assessed for eligibility, and data from papers considered eligible were extracted and used to create a table. This study was prospectively registered on PROSPERO under the ID "CRD42024511723." The MESH keywords used included "VTE," "thromboembolism," "cancer," "prophylaxis," "malignancy," and "thrombosis."

Only randomized controlled trials (RCTs) were included in the analysis. Cross-over designs, controlled clinical trials, and (historical) cohort studies were excluded. In instances where trials involved a mixed population, such as patients with various hematological malignancies, only the data specific to the ALL subgroup were extracted and utilized. The selected RCTs investigated the effectiveness of thromboprophylaxis measures during primary ALL treatment, with a focus on comparing the incidence of VTE in patients receiving specific thromboprophylaxis interventions against those in the control groups.

Data Extraction and Quality Appraisal

Three review authors independently extracted data from eligible studies using a standardized form. This included general study characteristics like ID, authors, journal/source, publication date, and funding sources, as well as specific study details such as design, setting, and intervention specifics like dosage and compliance. Participant characteristics such as demographics and medical history were all recorded. Interventions, including thromboprophylaxis, were documented in terms of type, frequency, and duration. Outcomes covering VTE definitions and incidence and adverse events were analyzed.

Methodological quality was assessed using a bias assessment form in line with the Cochrane Handbook for Systematic Reviews of Interventions [8], covering domains such as selection, blinding, randomization, incomplete data, reporting biases, and other potential sources of bias. Each item will be categorized as "low risk," "high risk," or "unclear risk."

Statistical Analysis

Measures of treatment effect were expressed based on the outcome type. ORs and relative risk (risk ratio) were used to express the outcomes of interest, including those related to VTE incidence and risk, as well as outcomes associated with adverse events such as bleeding events. Only RCTs with individual randomization were included, and VTE incidence was expressed as relative incidence rates among patients. The random-effects model was employed for the meta-analysis. Heterogeneity was assessed using chi-square tests and I^2^ statistics. The analysis was conducted using Review Manager version 5.4 (Cochrane Collaboration) [9].

Results

General Characteristics

**Figure 1:**
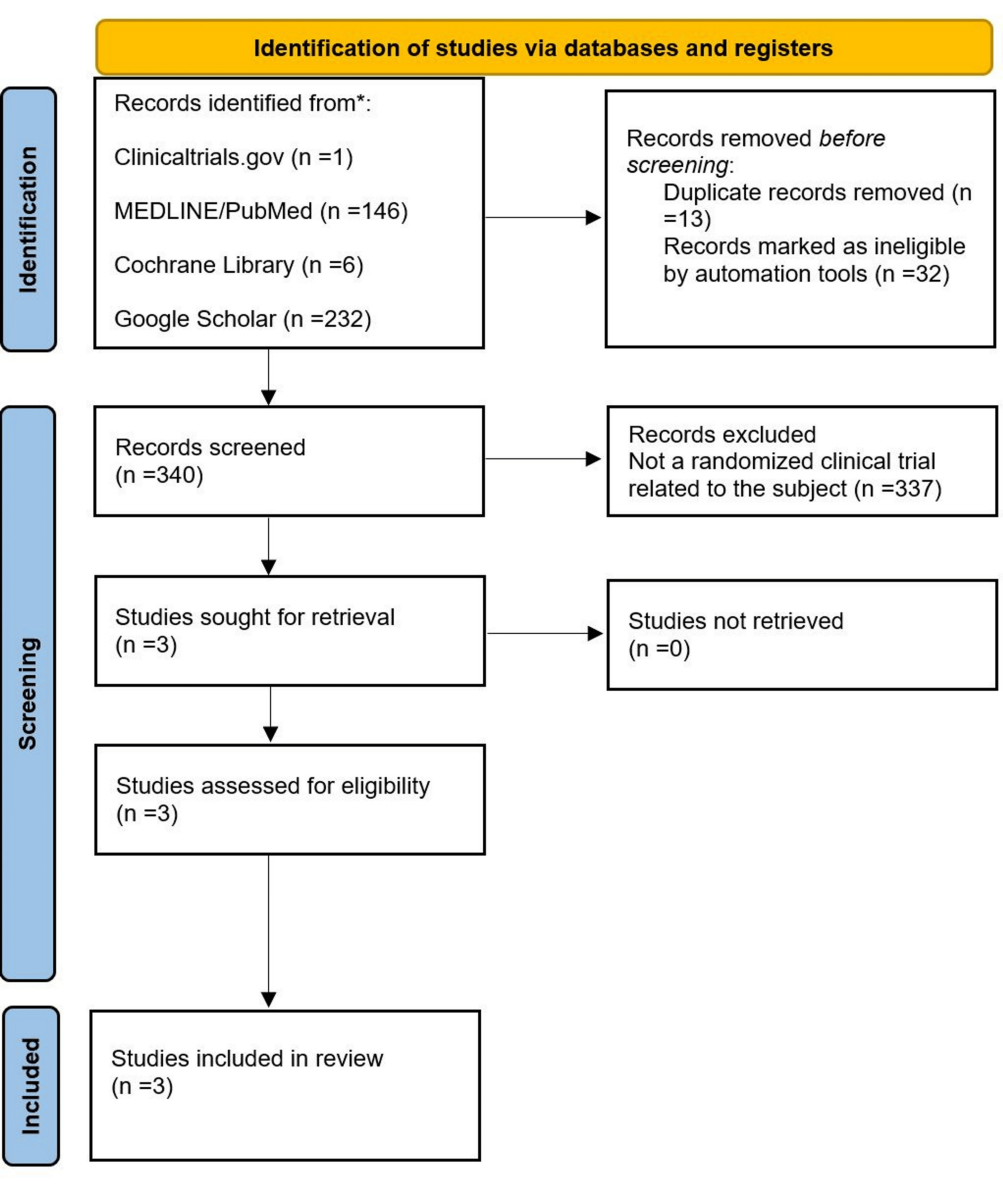
Preferred Reporting Items for Systematic Reviews and Meta-Analyses (PRISMA) flowchart for the search process.

The initial search yielded the following: one study from Clinicaltrials.gov, 146 potential articles from MEDLINE/PubMed, six potential articles from Cochrane Library, and 232 potential articles from Google Scholar. After we applied the exclusion criteria, only three of the papers found were deemed eligible as original RCTs, and these were the ones used in the meta-analysis. The first study by O’Brien et al. [10] explores the role of apixaban in thrombophylaxis in patients being treated for ALL. The second study by Griener et al. [11] explores the role of enoxaparin (low-molecular-weight heparin (LMWH)) and antithrombin in thrombophylaxis in patients being treated for ALL. The two groups in this study will be treated as separate entities during the analysis of the pooled data. The last study by Mitchell et al. [12] explores the role of antithrombin in thrombophylaxis in patients being treated for ALL. Figure 1 shows the search process according to the PRISMA statement. Table 1 shows an overview of the included studies.

**Table 1:** Overview of the included studies.

Author	Patient population	Thrombophylaxis regimen and dosage	Type of chemotherapy used	Phase of chemotherapy	Gender	Mean age
O'Brien, et al. 2023 [10]	Total number: 512 patients 256 patients received apixaban, 256 patients received standard of care.	Apixaban vs. standard of care: Apixaban dosage 2.5 mg twice daily for weight ≤35 kg .For patients above the mentioned weight, the dose was adjusted accordingly and it was given twice daily. Standard of care did not use systemic anticoagulation.	3/4 drug induction phase-based therapy with *E. coli* L-asp 6000 U/m2	Induction phase	Apixaban goup: 115 females (44.9%), 141 males (55.1%) Standard-of-care group: 107 females (41.8%), 149 males (58.2%)	Apixaban group: Mean age is 7.2 years ± 4.8 Standard-of-care group: Mean age 7.1 years ± 4.39
Greiner, et al. 2019 [11]	Total number: 949 patients 312 patients received standard of care; 317 patients received enoxaparin; 320 patients received antithrombin	Three groups: enoxaparin regimen, antithrombin regimen, and standard of care Enoxaparin group: Clexane 80-100 IU/Kg once daily subcutaneously. Antithrombi (AT) group: Dose was calculated as follows: [antithrombin target 100% – antithrombin actual] × kg body weight targeting at 100% AT activity *Was given when AT was below 80%* Standard-of-care group: Unfractionated heparin 2 IU/kg/hour As long as the infusion drip was running.	An induction protocol with prednisone or dexamethasone, vincristine, daunomycin, *PEG*-L-*asparaginase* (2 doses), and intrathecal methotrexate	Induction phase	537 males (56.6%), 412 females (43.4%)	Enoxaparin group (n = 317): mean age is 6.14. ≤5 years: 157 (49.5%). 6-10 years: 68 (21.5%). ≥11 years: 92 (29.0%) Antithrombin group (n = 320): mean age is 6.16. ≤5 years: 158 (49.4%). 6-10 years: 68 (21.3%). ≥11 years: 94 (29.4%) Standard-of-care group (n = 312): mean age is 5.72. ≤5 years: 174 (55.8%). 6-10 years: 57 (18.3%). ≥11 years: 81 (25.9%)
Mitchell et al. 2003 [12]	Total number: 85 patients 25 patients received antithrombin; 60 patients received standard of care	Two groups: Antithrombin and Standard of Care Antithrombin group: Dose was calculated as follows, given once per week: Units required (IU) = ((desired - baseline AT levels) x weight in kg)/1.4 where the baseline AT functional levels were expressed as a percent of normal. Standard of care did not use AT.	Asparaginase-based therapy	Induction phase	Antithrombin group: 10 females (40%), 15 males (60%) Standard-of-care group: 23 females (38.3%), 37 males (61.7%)	Antithrombin group: mean age is 3.8 (1.6-17.2) Standard-of-care group: mean age is 5.9 (1.9-16.7)

Methodological Quality of the Studies

Based on the Cochrane Handbook for Systematic Reviews of Interventions [8], all included studies were included in a risk-of-bias assessment. Details of this assessment are shown in Table 2.

**Table 2:** Risk-of-bias assessment result.

Author	Randomization and allocation	Blinding	Incomplete outcome data	Outcome assessment blinding	Selective reporting	Other biases
O’Brien et al. [10]	Low risk of bias	High risk of bias	Low risk of bias	Low risk of bias	Low risk of bias	Low risk of bias
Griener et al. [11]	Low risk of bias	High risk of bias	Unclear risk of bias	Low risk of bias	Low risk of bias	Unclear risk of bias
Mitchell et al. [12]	Low risk of bias	High risk of bias	Low risk of bias	Low risk of bias	Low risk of bias	Low risk of bias

In the study conducted by O’Brien et al. [10], the trial was open-label, so participants and personnel knew which treatment they were receiving. This presents a high risk of bias, especially for subjective outcomes like bleeding events. In the study conducted by Griener et al. [11], the study acknowledges the limitation of not blinding the antithrombotic intervention due to practical reasons. Blinding would have necessitated administering placebo injections to all groups, which was deemed impractical. While this decision introduces a high risk of bias, the reasoning is understandable given the challenges of blinding in a pediatric population.

In the study conducted by Mitchell et al. [12], the study was open, so attending physicians, nurses, and other healthcare providers were aware of treatment allocation. Blinding was considered impractical due to ethical concerns about administering placebos. However, outcome measures were assessed blinded by committees not involved in patient care, suggesting a moderate to high risk of bias.

Pooled Studies' Meta-Analysis

**Figure 2:**
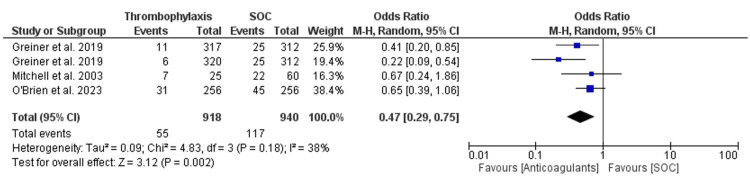
OR forest plot for the pooled studies' VTE event outcome. OR: odds ratio, VTE: venous thromboembolism References: O' Brien et al. [10], Griener et al. [11], Mitchell et al. [12]

**Figure 3:**
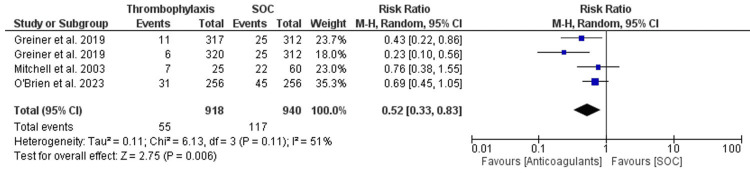
RR forest plot for the pooled studies' VTE event outcome. RR: relative risk, VTE: venous thromboembolism References; O' Brien et al. [10], Griener et al. [11], Mitchell et al. [12]

**Figure 4:**
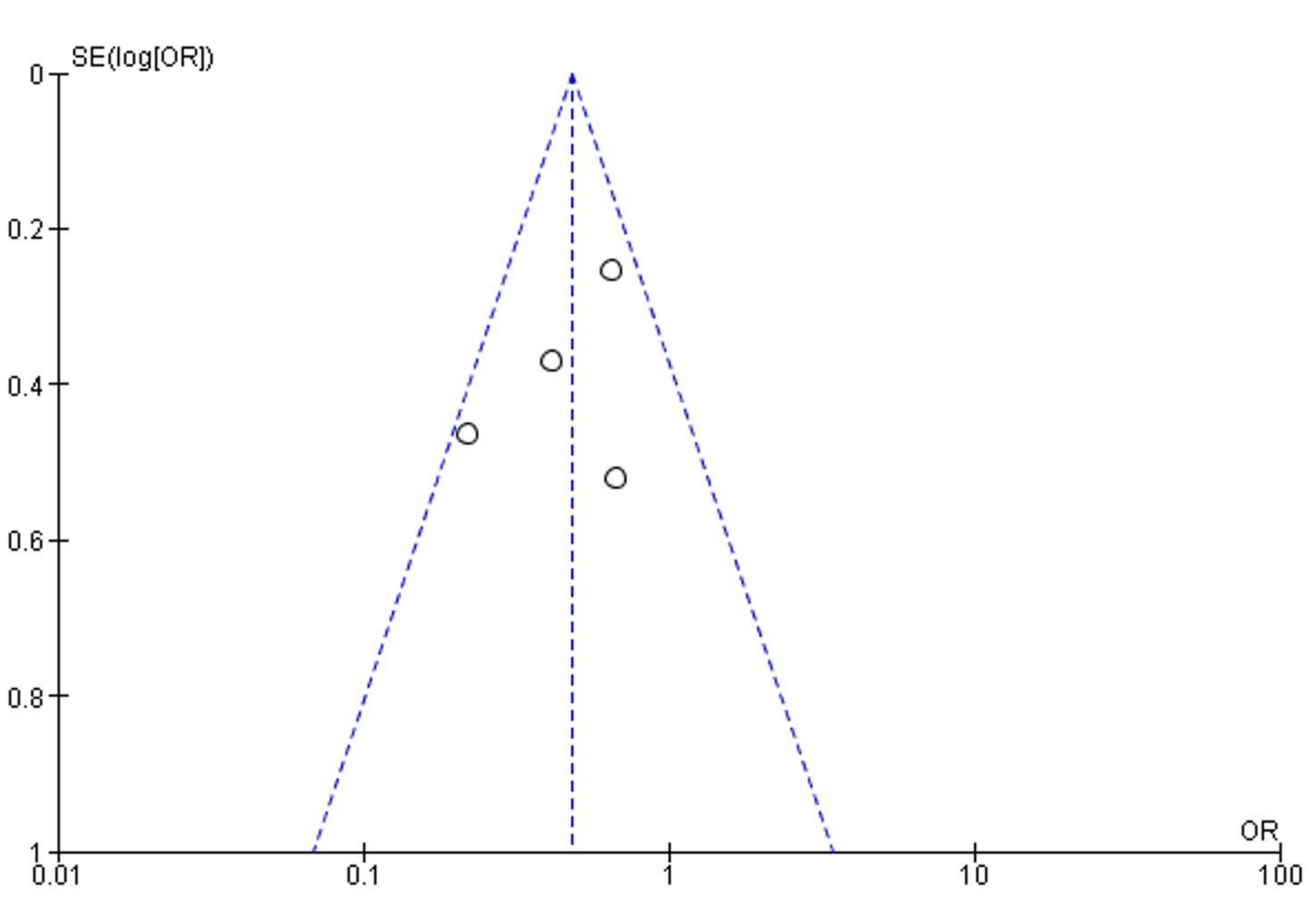
OR funnel plot for the pooled studies' VTE event outcome. OR: odds ratio, VTE: venous thromboembolism

The overall meta-analysis that was conducted for the pooled data from all the three studies follows a random-effects model to account for the expected differences between the studies. The OR for the outcome of VTE was calculated and was found to be 0.47 (95% CI 0.29-0.75). The overall heterogeneity of the OR for the outcome of VTE was calculated using both a chi-square analysis and I^2^ statistics and was found to be 4.83 (p-value = 0.18) and 38%, respectively. The overall effect was found to be Z = 3.12 (p-value = 0.002). The RR for the outcome of VTE was calculated and found to be 0.52 (95% CI 0.33-0.83). The overall heterogeneity of the RR for the outcome of VTE was calculated using both chi-square analysis and I^2^ statistics and was found to be 6.13 (p-value = 0.11) and 51%, respectively. The overall effect was found to be Z = 2.75 (p-value = 0.006). Figure 2 and Figure 3 illustrate the findings. A funnel plot was produced for the OR of the VTE outcome, as shown in Figure 4.

**Figure 5:**
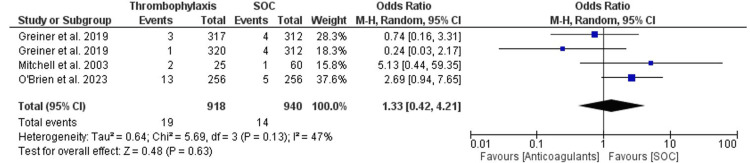
OR forest plot for the pooled studies' bleeding event outcome OR: odds ratio References: O' Brien et al. [10], Griener et al. [11], Mitchell et al. [12]

**Figure 6:**
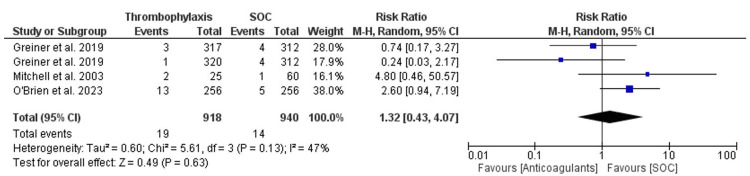
RR forest plot for the pooled studies' bleeding event outcome RR: relative risk References: O' Brien et al. [10], Griener et al. [11], Mitchell et al. [12]

**Figure 7:**
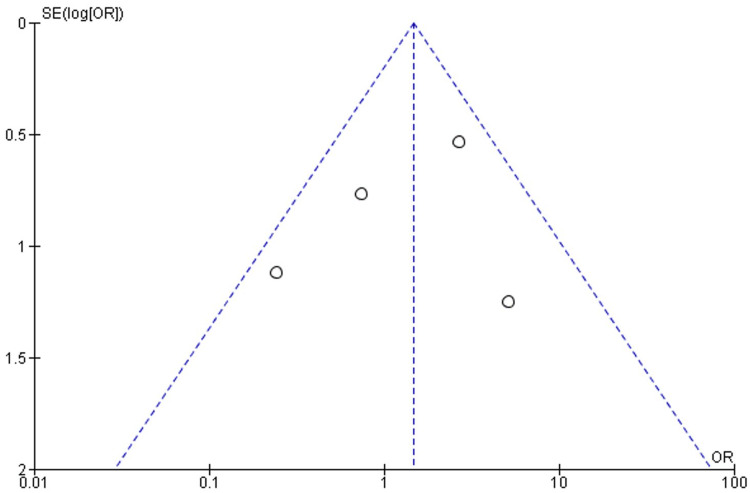
OR funnel plot for the pooled studies' bleeding event outcome OR: odds ratio

The OR for the outcome of bleeding was calculated and was found to be 1.33 (95% CI 0.42-4.21). The overall heterogeneity of the OR for the outcome of bleeding was calculated using both chi-square analysis and I^2^ statistics and was found to be 5.89 (p-value = 0.13) and 47%, respectively. The overall effect was found to be Z = 0.48 (p-value = 0.63), The RR for the outcome of bleeding was calculated and found to be 1.32 (95% CI 0.43-4.07). The overall heterogeneity of the RR for the outcome of bleeding was calculated using both chi-square analysis and I^2^ statistics and was found to be 5.61 (p-value = 0.13) and 47%, respectively. The overall effect was found to be Z = 0.49 (p-value = 0.63). Figure 5 and Figure 6 illustrate the findings. A funnel plot was also created for the OR of the bleeding outcome, as can be seen in Figure 7.

In the funnel plots for both VTE and bleeding outcomes, the distribution of included studies is evenly dispersed around the center line. This suggests that studies with both favorable and less favorable results have been included, indicating a low likelihood of publication bias. In addition, the funnel plots demonstrate that the precision of the effect estimates increases with larger study sizes, as smaller studies tend to scatter more widely.

Discussion

This systematic review and meta-analysis is the first to investigate the effectiveness of thromboprophylaxis in patients undergoing treatment for ALL. By synthesizing data from three RCTs, the study sheds light on the role of thromboprophylaxis measures, including antithrombin, apixaban, and enoxaparin, in reducing the incidence of VTE and improving treatment outcomes in this patient population.

This study demonstrated that individuals who received thromboprophylaxis measures, such as apixaban, enoxaparin, and antithrombin, exhibited decreased odds of developing VTE compared to those subjected to the standard of care (SOC). This disparity in the odds of VTE incidence can be attributed to the distinctive characteristics of these medications. For example, apixaban and enoxaparin boast a wide therapeutic index, rendering them not only more convenient for physicians to administer as prophylactic treatments but also offering a superior safety profile and more predictable pharmacokinetics and pharmacodynamics in contrast to traditional anticoagulants [13,14]. In addition, in the context of VTEs, particularly the general patient population not limited to patients with ALL, rhe AVERT trial [15] demonstrated that apixaban significantly reduced the risk of VTEs in cancer patients, achieving a 59.1% relative risk reduction compared to placebo, highlighting its effectiveness in lowering VTE incidence. Regarding the utilization of enoxaparin and antithrombin as thromboprophylaxis measures, research has indicated a significant reduction in the incidence of VTE with only a slight elevation in the risk of bleeding [16]. This is supported by a study conducted by Sibai et al. [17], where it was seen that the adoption of weight-adjusted enoxaparin led to a decrease in symptomatic VTE occurrences, while the frequency of bleeding events remained relatively unchanged.

The use of thromboprophylaxis agents in patients undergoing treatment for ALL highlights several important clinical outcomes. Sustained anticoagulation over time significantly reduces the case-fatality rate associated with recurrent thromboembolism without leading to a marked increase in major bleeding events, an essential balance in managing high-risk patients. However, individuals initially presenting with PE or provoked VTE show an increased rate of major bleeding, particularly early in treatment. This is especially concerning in the ALL population, where factors like thrombocytopenia and chemotherapy-related complications heighten the bleeding risk. In addition, shortened activated partial thromboplastin time (aPTT) values have been found to predict the likelihood of VTE recurrence after discontinuing oral anticoagulants, highlighting the need for careful monitoring of coagulation parameters. Abnormal aPTT values may indicate a higher risk of recurrence, suggesting that adjustments in anticoagulation therapy could be necessary to strike the right balance between preventing thromboembolism and avoiding bleeding complications [18].

In addition to the bleeding events, VTE recurrence remains a big obstacle when it comes to clinical management. A study conducted by Corentin Orvain et al. [4] showed that despite the encouraging outcomes observed, the use of antithrombin for ALL patients undergoing L-asparaginase therapy had persistent VTE occurrences despite thromboprophylaxis measures, and in the case of heparin, an increased risk of VTE was noted within their patient cohort. This could potentially be attributed to the characteristics of the patient population selected in these studies, as they may have had an elevated risk for thrombosis. Nevertheless, such studies underscore the necessity for prospective RCTs to reassess the role of such medications in the management of ALL patients undergoing chemotherapy.

Moving on, cost constitutes a pivotal aspect of healthcare management; therefore, the cost-effectiveness of these regimens is important for decision-making. Apixaban has emerged as the most cost-effective treatment method for VTEs among the three medications studied in this paper, with an average annual cost of approximately $40,000 USD (2023) in various scenarios, showcasing superior cost-effectiveness compared to using LMWH such as enoxaparin [19]. While antithrombin and apixaban exhibit relatively similar costs, apixaban typically demonstrates slightly better cost-effectiveness (saving approximately $65 USD (2023) per patient) and an approximate gain of 0.008 quality-adjusted life years (QALYs) [20].

Our study aligns with a 2020 study that recommends the use of LMWH for thromboprophylaxis during the induction phase of ALL therapy, particularly when asparaginase is included. For antithrombin prophylaxis, they suggest regular monitoring of antithrombin levels throughout asparaginase therapy. These recommendations are consistent with our findings. However, while they propose administering antithrombin concentrate when levels fall below 50% to 60%, the study in our meta-analysis used a threshold of 80%. As for apixaban, their study had insufficient data at the time to make definitive recommendations. By contrast, our study demonstrates significant benefits associated with apixaban for thromboprophylaxis and supports its use [21].

Despite the promising findings, the study has several limitations that warrant consideration. The open-label nature of some trials and the lack of blinding in others introduce potential biases that may influence the interpretation of results, particularly for subjective outcomes such as bleeding events. In addition, the heterogeneity observed among the included studies may limit the generalizability of findings and necessitate cautious interpretation. Furthermore, while the meta-analysis provides valuable insights into the effectiveness of thromboprophylaxis in reducing VTE incidence during ALL therapy, it is important to acknowledge the need for further research to address remaining uncertainties and optimize thromboprophylaxis strategies in this patient population. Future studies should focus on addressing methodological limitations, exploring the comparative effectiveness of different thromboprophylaxis agents, and identifying patient subgroups that may benefit most from thromboprophylaxis.

## Conclusions

This systematic review is the first of its kind to study effectiveness of thromboprophylaxis in reducing VTE risk during ALL treatment. The results indicate a significant decrease in VTE incidence with prophylactic interventions like apixaban, enoxaparin, and antithrombin compared to standard care. While acknowledging study limitations, including biases and heterogeneity, these findings underscore the potential of tailored thromboprophylaxis strategies to enhance patient safety and treatment outcomes in ALL. Further research is needed to optimize these approaches and address remaining uncertainties.
